# Enzymatic cellulose oxidation is linked to lignin by long-range electron transfer

**DOI:** 10.1038/srep18561

**Published:** 2015-12-21

**Authors:** Bjørge Westereng, David Cannella, Jane Wittrup Agger, Henning Jørgensen, Mogens Larsen Andersen, Vincent G.H. Eijsink, Claus Felby

**Affiliations:** 1 Department of Chemistry, Biotechnology and Food Science, Norwegian University of Life Sciences, 1432 Ås, Norway; 2 University of Copenhagen, Faculty of Science, Department of Geoscience and Natural Resources Rolighedsvej 23, 1958 Frederiksberg C, Denmark; 3 University of Copenhagen, Faculty of Science, Department of Food Science Rolighedsvej 30, 1958 Frederiksberg C, Denmark; 4 Department of Chemical and Biochemical Engineering, Technical University of Denmark, Søltofts Plads, 2800 Lyngby, Denmark

## Abstract

Enzymatic oxidation of cell wall polysaccharides by lytic polysaccharide monooxygenases (LPMOs) plays a pivotal role in the degradation of plant biomass. While experiments have shown that LPMOs are copper dependent enzymes requiring an electron donor, the mechanism and origin of the electron supply in biological systems are only partly understood. We show here that insoluble high molecular weight lignin functions as a reservoir of electrons facilitating LPMO activity. The electrons are donated to the enzyme by long-range electron transfer involving soluble low molecular weight lignins present in plant cell walls. Electron transfer was confirmed by electron paramagnetic resonance spectroscopy showing that LPMO activity on cellulose changes the level of unpaired electrons in the lignin. The discovery of a long-range electron transfer mechanism links the biodegradation of cellulose and lignin and sheds new light on how oxidative enzymes present in plant degraders may act in concert.

While it has been known for decades that enzymatic oxidation of lignin by laccases and peroxidases plays a role in microbial biomass conversion of lignin, it has only very recently become apparent that oxidative processes also play a major role in the conversion of polysaccharides. The latter process is carried out by so-called Lytic Polysaccharide MonoOxygenases (LPMOs)^1^, which are copper-dependent enzymes capable of breaking glycosidic bonds in polysaccharides, such as cellulose, xyloglucan, glucomannan, xylan, starch and chitin^2^,^3^,^4^,^5^,^6^,^7^,^8^,^9^. LPMO activity depends on the presence of molecular oxygen and requires an electron donor. So far, it has been shown that electrons can be provided by cellobiose dehydrogenase (CDH)^10^ or by small molecule electron donors such as ascorbic acid^7^ or gallic acid^6^. However, virtually nothing is known about how the LPMO-catalyzed redox reactions and electron transfers function within the plant cell wall matrix during biological decay.

During biomass conversion by fungi, many of the lignin- and carbohydrate- active redox enzymes are expressed simultaneously with hydrolytic enzymes^11^,^12^, which points to a possible interplay between these enzyme systems. LPMO-encoding genes are abundant in the genomes of biomass degrading and plant pathogenic fungi, and when grown on lignocellulosic material, LPMOs are among the most highly expressed proteins^13^,^14^,^15^,^16^. Notably, there is ample evidence that LPMOs enhance the power of the fungal degradative enzyme machinery^17^,^18^. Today, LPMOs are important components in industrial enzyme cocktails used for saccharification of cellulosic biofuel feedstocks^19^,^20^.

LPMOs are copper enzymes^6^, which cycle between Cu (I) and Cu (II) to activate molecular oxygen. Kim *et al.* (2014) suggested a mechanism involving the formation of a copper-oxyl radical that abstracts a hydrogen and then hydroxylates the substrate via an oxygen-rebound mechanism^21^. The details of oxygen activation were further elaborated by X-ray absorption studies of the active site copper, leading to the conclusion that the initial oxygen species is a super oxide^22^. During *in vivo* conditions, the electron donor may be CDH, but microorganisms may also utilize other approaches for providing electron donors to oxidative reactions. Lignin is one of the main structural components in plants and has an electron configuration that provides a low barrier for electron transfer. There are indications that lignin may act as electron donor for LPMOs^18^,^23^, but substantial evidence is scarce.

One-electron transfer from lignin, proceeding via an outer sphere mechanism^24^, is well known and has been described for lignin oxidizing enzymes such as laccases^25^. The transfer may take place through direct interactions between substrate and enzyme or through long-range transfer involving low molecular weight electron shuttles. Using wood substrates, it has been shown that the lignin polymer can be oxidized by laccases via an electron transfer process where low molecular weight lignin compounds act as shuttles or mediators between the enzyme and the polymer^26^. Several other studies published in the 1990s demonstrate mediator-based laccase action including studies on laccase-catalyzed delignification in the pulp and paper industry^27^,^28^. Notably, the interactions between low- and high-molecular weight lignin compounds stabilize the population of unpaired electrons in the low molecular weight lignin fraction, which favors electron transfer reactions^29^.

The function of electron shuttles for *in vivo* oxidation of lignin was suggested also to involve the aromatic acid metabolite 3-hydroxyanthranilic acid (3HAA) secreted by the white rot fungus *Pycnoporus cinnabarinus*^30^. However, in a subsequent study, it was unambiguously shown that laccases do not utilize 3HAA for lignin depolymerization^31^. The role of this compound in oxidative reactions is thus still enigmatic and could perhaps relate to LPMO function, as discussed below.

In this work, we demonstrate how LPMO-catalyzed oxidation of cellulose in a plant cell wall matrix is linked to lignin via long-range electron transfer from the lignin polymer to the active site of the LPMO. We show that a wide variety of small molecules found in biomasses, including microbial metabolites such as 3HAA, can act as electron donors for the LPMO. Oxidized cellulose products were analyzed by HPAEC^32^ and MALDI-ToF mass spectrometry, whereas low and high molecular weight lignin fractions were characterized by ESI-MS and gel permeation chromatography, respectively. Using Electron Paramagnetic Resonance (EPR) spectroscopy, we show that the electron transfer process results in conspicuous changes in the population of unpaired electrons in the lignin polymer. Our findings link cellulose and lignin conversion and point towards new interactions and synergies in microbial plant degradation.

## Results

### Oxidation of cellulose to oligosaccharides

**Figure 1:**
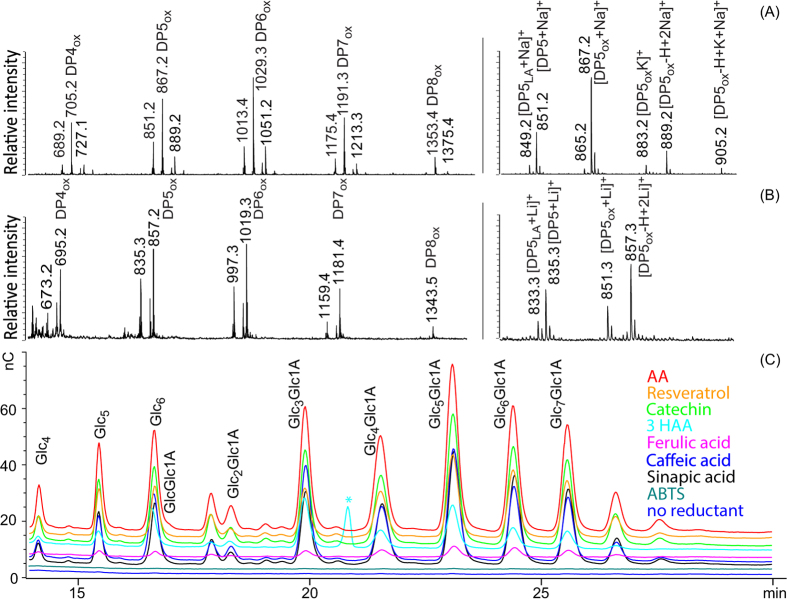
The activity of *Tt*LPMO9E on PASC in the presence of various electron donors. (A) MALDI-ToF spectrum of soluble products. The most prominent masses in the spectrum represent the sodium adducts, [M+Na]^+^, of the aldonic acids, which are annotated with DP_n_ox and the *m/z* (DP5ox = Glc_4_Glc1A). All ion clusters have a similar adduct distribution as shown for the DP5 cluster in the zoom in picture to the right. Note that the cluster includes the native pentamer and the lactone form of the oxidized pentamer (“LA”). (**B**) MALDI ToF spectrum obtained after lithium doping of the samples for further validation of the product annotations of panel A. All ion clusters have a similar adduct distribution as shown for the DP5 cluster in the zoom in picture to the right; notably, after lithium doping Na and K-adducts are no longer present. The presence of double adducts, as seen in panels A and B, is indicative of the presence of a carboxyl group as in an aldonic acid^51^. The reaction conditions were: 1.5% (w/v) PASC, 1μM *Tt*LPMO9E, 25 mM Bis-Tris pH 6.5, 2 mM ascorbic acid, incubation for three days at 50 °C, at 1000 rpm. (**C**) HPAEC chromatograms showing products obtained after PASC degradation in the presence of a variety of electron donors. The conditions were: 2 mM electron donor, 1 μM *Tt*LPMO9E, 1.5% (w/v) PASC, 25 mM Bis-Tris pH 7.0, incubation for 13 hours at 50 °C, 1000 rpm. The electron donors used were: ascorbic acid (AA), resveratrol, catechin, 3-hydroxyanthranilic acid (3HAA), ferulic acid, caffeic acid, sinapic acid, ABTS (2, 2′-azinobis(3-ethylbenzthiazoline-6-sulfonate). Note that there are no products in the samples from the ABTS reaction and the negative control, i.e the reaction without added reductant (LPMO-reductant). Peaks are labeled using Glc for glucose and Glc1A for gluconic acid. The peak labeled with an asterisk represents a background signal from 3HAA also present in the control without LPMO.

LPMOs acting on cellulose show varying regioselectivities. Some exclusively oxidize C1, others exclusively oxidize C4, whereas some oxidize both C1 and C4^33^. MALDI-ToF MS (Fig. 1A,B) and HPAEC (Fig. 1C) of products generated from PASC show that the previously uncharacterized *Tt*LPMO9E generates C1-oxidized products, i.e. lactones that are in equilibrium with the (dominating) aldonic acid form. The product profiles shown in Fig. 1 are very similar to those previously observed for strictly C1 oxidizing *Pc*LPMO9D^34^. The use of C1 oxidizing LPMOs was essential in this study since the aldonic acid products generated by such LPMOs are easier to detect than C4 oxidized products^35^. While LPMO activity on soluble cellodextrins has recently been demonstrated for a C4 oxidizing LPMO^35^, we could not detect such activity for *Tt*LPMO9E nor *Pc*LPMO9D (results not shown).

Fig. 1C shows the importance of an electron donor for LPMO action. We tested a series of potential electron donors, several of which have not been used previously to activate LPMOs. The results show that ascorbic acid works well and that many other electron donors work, albeit with varying efficiencies (Fig. 1C). Resveratrol, catechin, caffeic acid, sinapic acid, and also hydroquinone (Fig. S1), worked well, whereas ferulic acid was less efficient. ABTS, a well-known laccase mediator, was not able to donate electrons and thus activate the LPMO (Fig. 1C). The formal reduction potentials of ABTS (0.67 and 1.08 V^36^) are much higher than those observed for LPMOs (0.25–0.3 V), which explains why no reduction of Cu(II) and concomitant LPMO catalytic activity were observed. Fig. 1C also shows that 3HAA, a low molecular weight metabolite secreted by the white rot fungus *Pycnoporus cinnabarinus* activates the LPMO.

**Figure 2:**
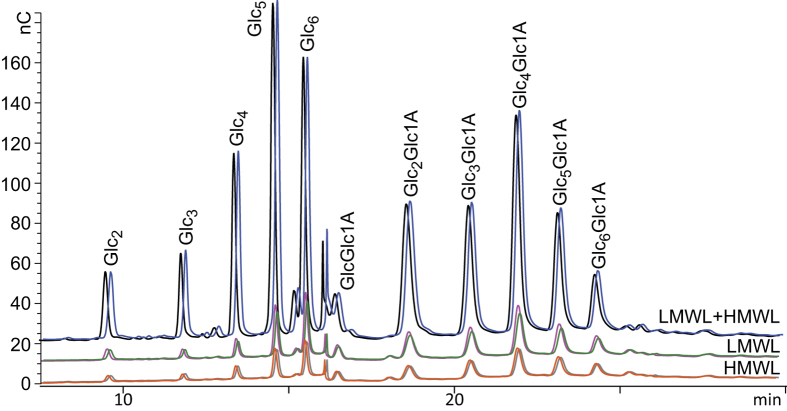
The activity of *Tt*LPMO9E on PASC in the presence of lignin fractions. The chromatograms show oligosaccharides released from PASC upon treatment with *Tt*LPMO9E in the absence of an added electron donor, but in the presence of various lignin fractions (duplicate reactions are shown with a slight time offset): orange and grey, organosolv HMWL from pretreated wheat straw (PWS); green and magenta, extracted LMWL from PWS; blue and black, mixture of LMWL and organosolv HMWL from PWS. Conditions (200 μL total reaction volume): 1 μM *Tt*LPMO9E, 50 ˚C, 1000 rpm, 0.75% PASC, 1 mg HMWL, 20 μL LMWL (corresponding to a final concentration of appr. 2 mM), 12 hours reaction time. The highest product levels shown in this figure correspond to roughly 50% of the maximal levels obtained after running reactions to completion.

Reactions containing individual lignin fractions, either soluble low- or insoluble high-molecular weight lignin (LMWL and HMWL, respectively) showed very low or no LPMO activity (Fig. 2). However, if LMWL and HMWL were added together, product levels increased drastically (Fig. 2). Similar effects were also observed for *Pc*LPMO9D, another C1-oxidizing LPMO (Fig. S2). All tested combinations of LMWL and different HMWLs (hydrolysis lignin, organosolv lignin or Klason lignin; see Methods section) showed similar synergistic effects on LPMO activity. On the other hand, when HMWL were tested together with small molecules from Fig. 1 like e.g. hydroquinone and 3HAA (Fig. S1) instead of LMWL, no synergy was observed as the level of oxidized products did not increase.

### Unpaired electron populations in lignin fractions

**Figure 3:**
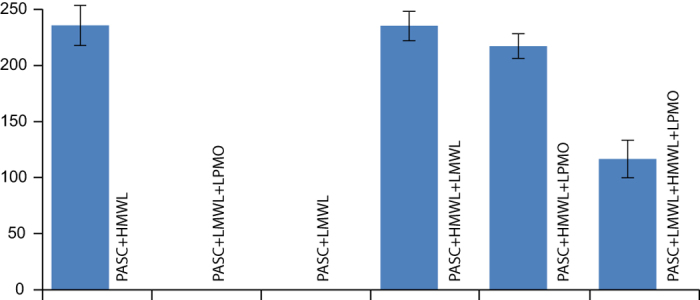
Relative levels of unpaired electrons in different reaction mixtures. Reaction were set up as described in the legend of Fig. 2. After the reactions, free electrons were quantified using EPR spectroscopy. Standard deviations for each experiment are given as vertical bars (n =3).

Electron Paramagnetic Resonance (EPR) was used to unveil the nature of unpaired electrons (radicals) in the system, and how their levels were affected by the redox reactions taking place. After incubation of reaction mixtures, the suspensions containing PASC, enzyme, products and various lignin fractions were transferred to EPR tubes, frozen in liquid nitrogen and subjected to EPR spectroscopy as described in the Methods section. Unpaired electrons with a g-value of 2.0035 were detected in samples containing high molecular weight lignin fractions only. The g-value is consistent with delocalized unpaired electrons in an aromatic carbon macromolecule^37^. No fine structures in the EPR signal could be seen, which is typical for macromolecules. Unpaired electrons could not be detected in samples containing various combinations of enzyme, PASC and LMWL, but were detected in all samples containing HMWL (Fig. 3). The highest level of unpaired electrons was found in the samples containing HMWL and incubated under conditions where there was no LPMO activity (i.e. no enzyme or LMWL added). LPMO activity in the presence of LMWL and HMWL led to a reduction in the level of unpaired electrons (Fig. 3). This may indicate that crosslinking reactions between low- and high-molecular weight lignin are taking place^38^, as discussed further below.

### Structural evaluation of LMWL and HMWL

**Figure 4:**
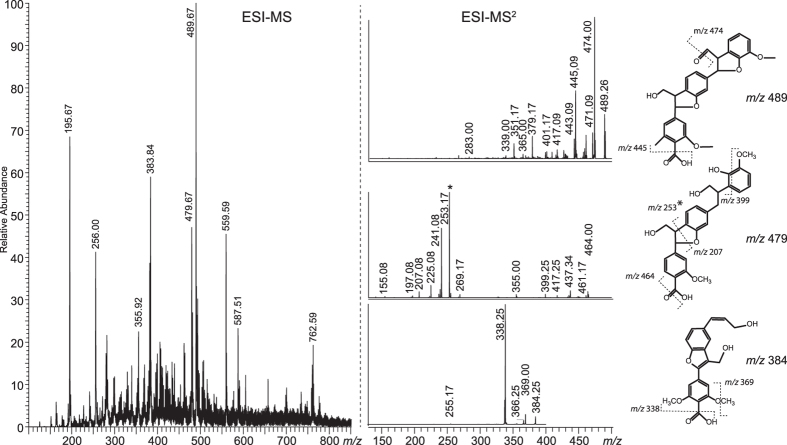
ESI-MS analysis of the LMWL lignin extracted from PWS. The ESI-MS spectrum to the left shows ions corresponding to dimeric and trimeric phenolic units. The spectra to the right show fragmentation patterns for major peaks, supporting that these are lignin derivatives, either trimers (*m/z* 489, upper spectrum, and *m/z* 479, middle spectrum) or dimers (*m/z* 384, lower spectrum). The quite abundant *m/z* 559 peak showed a fragmentation pattern similar to that of the *m/z* 479 peak. The structures shown to the right are based on known substructures in the lignin macromolecule and are just meant as examples of molecules that could give the observed mass spectra. The predominant *m/z* 253 fragment (denoted *) may arise from dehydration during the fragmentation.

Fig. 4 shows an analysis of the LMWL fraction obtained from pretreated wheat straw (PWS). The MS spectra of the LMWL show a range of ions with *m*/*z* values corresponding to trimeric/dimeric lignin-derived compounds. No exact determination of structures was done, but it was established that the *m/z* values of the dominating ions correspond to structures typical for lignin substructures^39^. Fragmentation patterns for the most prominent peaks and possible lignin sub-structures compatible with these patterns are presented in Fig 4. These possible dimers and trimers are highly conjugated, stabilizing the unpaired electron by delocalization between the pi-orbitals.

**Figure 5:**
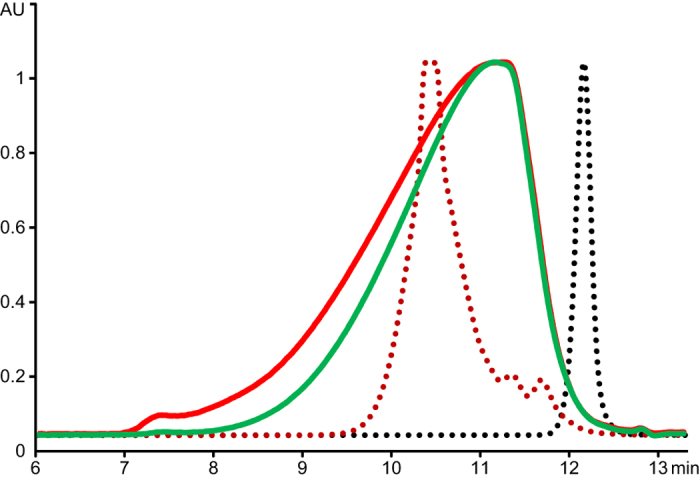
GPC chromatograms of organosolv HMWL lignin. Green, HMWL from pretreated wheat straw; red, the same HMWL after incubation with cellulose, LMWL and LPMO; black dotted line, phenol (94 Da); red dotted line, tannic acid (1701 Da). Comparison of the red and green chromatograms shows that the molecular weight distribution of the HMWL increases following the LPMO reaction probably due to crosslinking with LMWL. HMWL incubated with cellulose and LMWL, but without the LPMO yielded a chromatogram identical to the green chromatogram shown here.

Gel permeation chromatography of the HMWL derived from PWS by organosolv extraction (Fig. 5) showed a broad distribution peak which is typical for HMWL under these chromatographic conditions^40^. Practically no compounds of low molecular weight can be seen in the HMWL fraction. Importantly, lignin obtained from samples after incubation with LPMO, cellulose, HMWL and LMWL displayed a shift towards higher molecular weight (Fig. 5) and a slightly larger peak area compared to samples with LPMO, cellulose and HMWL. This possibly reflects crosslinking between LMWL and HMWL. Control experiments without LPMO present did not show this increase in the molecular weight.

## Discussion

The most important result of this study is the demonstration of the synergy between LMWL and HMWL in boosting LPMO activity. Individually, the two different lignin fractions had very little, if any, effect on LPMO activity, but when combined, the enzyme activity was similar to that observed with the most efficient of other reductants (Fig. 2; Fig. S2). Electron carriers like hydroquinone and 3HAA, which are efficient electron donors for LPMOs, did not appear to show synergistic effects with HMWL (Fig. S1), probably because of less delocalized electrons, disproportionation is favored rather than electron transfer. The results point to a possible cyclic mechanism where water-soluble LMWL compounds shuttle electrons from HMWL to the LPMO active site. Besides organosolv lignin, also mildly prepared hydrolysis lignin with a structure close to native lignin (data not shown) and Klason lignin prepared from wheat straw (Fig. S2B) were used as HMWL, showing similar synergistic effects. This indicates that the synergistic effect of HMWL and LMWL is generic for lignin structures.

**Figure 6:**
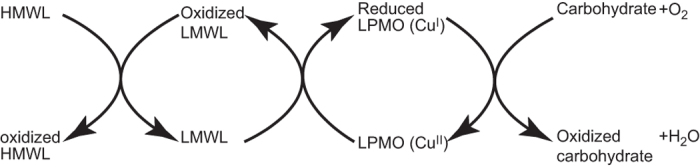
Suggested electron transfer cycle between lignin and the active site copper of the LPMO.

Electron shuttling between lignin polymers and oligomers has previously been demonstrated in connection with oxidation of lignin by laccases^26^,^41^. However, little attention has been paid to the importance of the low molecular weight lignin structures. Some stabilization of the unpaired electron will be necessary to enable the shuttling mechanism to work over longer distances. At the same time, there should be a favorable difference in redox potential to drive the reaction from lignin to the LPMO active site. The molecular weight distribution of the LMWL shows that masses corresponding to dimers/trimers are dominating. Such structures with a cluster of pi-orbitals favor the stabilization of the unpaired electrons. The importance of having a polycyclic mediator like LMWL is supported by the lack of synergy when low molecular weight electron donors were used as electron donors (Fig. S1). Thus one could envisage a lignin-dependent cyclic electron donating mechanism as shown in Fig. 6.

The lignin polymer is an efficient electron donor with a naturally occurring population of unpaired electrons and the delocalization of these makes lignin highly stable also in an oxidized form^26^,^42^. We could not detect any increase in the population of unpaired electrons in the HMWL, as one perhaps would expect if electrons were being donated from the HMWL. Instead, the level of unpaired electrons decreased as a result of LMWL-mediated electron transfer to the LPMO (Fig. 3). An alternative explanation, supported by the observed increase in HMWL molecular weight upon LPMO action, entails that the oxidative reactions in the system lead to crosslinking between oxidized LMWL and HMWL. In other words, as the overall reaction proceeds, the oxidized LMWL may react with unpaired electrons in the HMWL, thereby decreasing the unpaired electron population in the HMWL.

As part of this study we also tested several small molecule electron donors that have not previously been assessed for their LPMO-boosting potential. Interestingly, we found that 3HAA from the wood degrading white rot fungus *Pycnoporus cinnabarinus* functions well as electron donor for the LPMO (Fig. 1B). In the recently published genome of *Pycnoporus cinnabarinus* there are 15 annotated LPMOs^43^. Considering the broad range of compounds known to activate LPMOs (e.g. Fig. 1), it is conceivable that white rot fungi utilize their own metabolites, such as 3HAA, for example in situations where lignin and other reductants are absent. Notably, besides oligomeric fragments of lignin (LMWL), also other compounds with possible effects on LPMO action are common in plants, including flavonoids, tannins and lignans^44^,^45^. While these compounds on the one hand may protect the plants against pathogens, they may also be used by degrading fungi as electron shuttles for their enzyme systems.

The discovery of a link between enzymatic cellulose oxidation and lignin via a long-range electron transfer mechanism sheds new light on our understanding of the redox chemistry applied by fungi and raises interesting questions for further research. Our data show that the redox state of lignin affects polysaccharide conversion through effects on LPMO efficiency. It is thus possible that redox modifications of lignin catalyzed by the laccases and peroxidases of wood degrading microorganisms are somehow coupled to the efficiency of polysaccharide conversion. Unravelling these possible connections, possibly also involving metabolities such as 3HAA, will generate important novel insights into fungal biomass degradation and may eventually lead to more efficient industrial processing of biomass.

## Methods

### Chemicals and enzymes

Ascorbic acid, hydroquinone, 3-hydroxyanthranilic acid (3HAA), resveratrol, catechin, caffeic acid, tannic acid, ferulic acid, sinapic acid, and ABTS (2, 2′-azinobis(3-ethylbenzthiazoline-6-sulfonate) were obtained from Sigma Aldrich (Saint Louis, USA). Stock solutions of 100 mM were made in either water (ascorbic acid, hydroquinone, ABTS and tannic acid) or pure ethanol (3HAA, resveratrol, catechin, caffeic acid, ferulic acid and sinapic acid) and kept at -20 °C in the dark. Commercial cellulase mixtures Celluclast 1.5L and Novozym188 were obtained from Novozymes A/S, Denmark. The Celluclast 1.5L mixture had a protein content of 127 mg/g, containing 62 FPU/g cellulase activity and 15 U/g β-glucosidase activity. Novozym188 had a protein content of 220 mg/g, containing 231 U/g β-glucosidase activity. Purified *Thielavia terrestris* LPMO (*Tt*LPMO9E, previously *Tt*GH61E^17^), was obtained from Novozymes A/S. *Pc*LPMO9D (previously known as *Pc*GH61D) from *Phanerochaete chrysosporium* was cloned and expressed in *Pichia pastoris* and purified according to^34^.

### Wheat straw hydrothermal pretreatment

Wheat straw (*Triticum aestivum* L.) was pretreated using a Parr reactor with 100 mL capacity. The hydrothermal pretreatment was conducted in an oil bath set at 194±1.5 °C with a residence time of 20 minutes, at 10% dry matter content in water, without addition of chemicals. A washing and pressing step was applied after the pretreatment, to remove solubilized sugars and degradation products (it has previously been shown that little LMWL is lost during this procedure^46^). The PWS was dried and grinded in a coffee blender for 5 minutes prior to further use. The chemical composition of the solid fraction was determined using a modified version of the NREL method for lignocellulose biomasses^20^, as: glucans 53%, xylans 4%, lignin 34%, and ash 6%.

### Microcrystalline cellulose PASC preparation

Avicel (microcrystalline cellulose Sigma Aldrich PH101) was swollen with phosphoric acid to generate phosphoric acid swollen cellulose (PASC) as previously described^47^ with a few modifications: 4 grams of Avicel were suspended in 100 mL of phosphoric acid (85% w/v) at 40 °C and magnetically stirred for 1 hour. The mixture was then poured into 1900 mL of water and kept at 40 °C with further stirring for 1 hour. The suspension was left stationary to allow the fibers to sediment before decanting the supernatant. The suspension was washed four times with 2 L H_2_O (MilliQ-quality), 2 times with 2 L of a 1% NaHCO_3_ solution to reduce acidity, and then three additional times with 2 L H_2_O (MilliQ-quality) and stored at 4 °C until further use. The final cellulose content of the PASC suspension, was determined by enzymatic hydrolysis (24 hours, 50 °C) with a high dosage of Celluclast 1.5L cellulolytic enzymes and Novozym 188, followed by the determination of released glucose, leading to an estimated cellulose content of 1.5% w/v. The average DP of the PASC was determined by measuring the total number of reducing ends^48^ and compare this to the total amount of monomeric glucose, giving a degree of polymerization of 52.

### Lignin preparation and isolation

Pretreated wheat straw was used for the lignin preparation, since it is generally accepted that only little modification of the native lignin structure occurs during this kind of treatment while still enabling efficient purification and extraction.

#### HMWL Klason lignin

Raw and pretreated wheat straw where subjected to sulfuric acid (4%) hydrolysis in order to remove all the carbohydrates present in the form of cellulose and hemicellulose. The procedure was based on the NREL protocol^49^. After polysaccharide hydrolysis and dissolution the resulting lignin was filtered, dried at 40 °C, powdered with a pestle and mortar, and then stored in the dark at 4 °C (like all other lignin fractions prepared below) until further use.

#### Hydrolysis lignin

PWS was hydrolyzed with excess of enzymes in order to remove all the cellulose and hemicellulose left after the hydrothermal pretreatment to isolate the lignin only. The enzymes used were a mixture of Celluclast 1.5L and Novozyme 188 at 5:1 ratio. The enzymatic hydrolysis was run with an enzyme loading of 75 FPU/gram dry PWS, at 50 °C for 72 hours in 50 mM sodium acetate buffer at 4.8 pH^50^. After the hydrolysis, the PWS was washed 3 times with distilled water at a PWS/water ratio of 1:100. The treatment was repeated once, after which the residual material was dried and powdered using pestle and mortar. Compositional analysis of the resulting material yielded 90% lignin, 6% ash and 2% glucans.

#### HMWL Organosolv lignin

PWS was suspended in an aqueous ethanol solution (50:50 water to ethanol) at a 5:1 liquid to solid ratio and heated at 220 °C in a 1L Parr reactor for 80 minutes. After cooking, the residue was filtered at 75 °C. Solubilized lignin was precipitated by adding water at three times the original amount and recovered by filtration. The lignin enriched fraction was dried (40 °C) and grinded with a pestle and mortar.

#### Extraction of Low molecular weight lignin (LMWL)

5 grams of PWS was suspended in 100 mL of a 90% ethanol/ 10% water mixture for low molecular weight lignin extraction. The incubation was done at room temperature for 1 hour and in the dark. The extracted lignin (supernatant after centrifugation), denoted LMWL, was collected and kept in the dark at 4 °C. Immediately prior to enzyme reactions the concentration of soluble lignin was approximately doubled by evaporation of ethanol at 40 °C in the dark. The concentration of condensed LMWL was 1% DM as determined by polymerization with laccase and subsequent precipitation. Laccase from *Trametes versicolor* (Sigma Aldrich) was added at a concentration of 3 U/mL to 10 mL LMWL for 4 hours at 50 °C. The pH was lowered to 3 by addition of 0.1 M HCl, and the precipitate was removed by centrifugation, dried and weighed.

### High-performance anion-exchange chromatography (HPAEC)

HPAEC was run on an ICS 5000 system, equipped with a PAD detector (Dionex, Sunnyvale, CA, USA) set up with a CarboPac PA1 column (2×50 mm guard column followed by a 2×250 mm analytical column) operated at a flow of 0.25 mL/min, at 30 °C. Chromatography was conducted as described in^32^. In short, elution involved a linear gradient from 100%A:0%B to 90%A:10%B (10 min), followed by an exponential gradient to 70%A:30%B (15 min), and lastly an exponential gradient to 100% B (5 min) (eluent A = 0.1M NaOH, B = 0.1M NaOH and 1M NaOAc).

### Enzymatic reactions

Standard experimental conditions for recording LPMO activity were: 1.5% w/v PASC, 2 mM ascorbic acid, 20 mM of citrate-phosphate buffer (pH 5.9), 0.05 mg/mL LPMO with 200 μL total reaction volume. Reactions were done in 2 mL Eppendorf tubes in an Eppendorf thermomixer operated at 1000 rpm and 50 °C. Other non-lignin compounds to be tested as potential reductants were added to a final concentration of 2 mM, replacing ascorbic acid. In reactions with lignin, ascorbic acid was omitted; HMWL was added to a final concentration of 5 mg/mL, whereas 20 μL LMWL was added. The final concentration of LMWL was approximately 2 mM assuming an average molecular weight of 300 Dalton. Control reactions with the same combinations of the lignins but without addition of enzymes were also conducted. Several HMWLs were tested; all were derived from pretreated wheat straw (PWS) but obtained through different isolation methods: Klason lignin, hydrolysis lignin and organosolv lignin.

### Electron paramagnetic resonance (EPR) spectroscopy

Enzymatic reactions for EPR analysis were proportionally the same as the enzymatic reactions described above but scaled to contain 7 mg dry HMWL/reaction, which was necessary to provide sufficient EPR signals. Following treatments, suspensions containing PASC, lignins and enzyme were transferred to 5 mm OD quartz tubes (710-SQ, Wilmad-Labglass, Vineland, NJ, USA), and frozen in liquid nitrogen in order to stop further reactions and to stabilize radicals in the liquid fraction. All steps of sample preparation to analysis were done under red light in a dark-room to eliminate any formation of unpaired electrons from UV-radiation^26^. The EPR spectra were recorded at 77 K using an ECS 106 X-band EPR spectrometer (Bruker, Karlsruhe, Germany), equipped with an X-band ER 4103TM cavity and a liquid nitrogen Dewar flask (819-Q, Wilmad-Labglass, Vineland, NJ, USA). The modulation amplitude was 1.02 G and the microwave attenuation 30 dB. Relative levels of unpaired electrons in the frozen suspensions were quantified by double integration of the first-derivative EPR signal. Samples from all treatments were prepared and measured in triplicate.

### Gel Permeation chromatography (GPC)

Solid fractions from lignin-containing enzyme reactions were dried overnight under high vacuum. Subsequently, the dried material was dissolved in a 9:1 DMSO:water mixture containing 0.05 M LiBr (the eluent), followed by sonication for 15 min. After centrifugation for 5 min at 12.000 rpm the supernatant was transferred to a sample vial. The GPC was performed isocratically with a Hitachi 7000 system set up with a PolarSil column (300 mm, 5 μm particles, 100 Å porosity from Polymer Standard Service) run at 1 mL/min and 40 °C. Detection was obtained using a UV detector (280 nm), using tannic acid and phenol as external standards.

### Direct infusion ESI-MS

The direct infusion analysis was performed on a linear iontrap VelosPro LTQ from ThermoScientific. The sample, enzymatic LMWL from PWS diluted 50 times in methanol, was delivered continuously to the spray by a syringe pump operating at 15 μL/min. The instrument was operated in negative mode with an ionization voltage of 1.5 kV, auxiliary and sheath gas settings of 55 and 10 respectively (arbitrary units) and with a capillary and source temperature of 275 °C and 250 °C, respectively. Full scan spectra were collected in the mass range from *m/z* 100-2000. MS2 was performed with CID fragmentation at a relative energy intensity of 45 and helium as the colliding gas. Data handling was done with Xcalibur 2.2 SP1.48 (ThermoScientific).

### MALDI-ToF MS

Two microliter of a saturated solution of 2,5- dihydroxybenzoic acid (DHB) in 30% acetonitrile was applied on an MTP 384 ground steel target plate TF (Bruker Daltonics). One microliter sample was then mixed with the DHB droplet and dried under a stream of air. The samples were analyzed with an Ultraflex MALDI-ToF/ToF instrument (Bruker Daltonics GmbH, Bremen, Germany) controlled by the FlexControl 3.3 software package. The instrument was operated in positive acquisition mode and the acquisition range used was from *m/z* 0 to 3000. The data were collected from averaging 250 laser shots, with the lowest laser energy necessary to obtain sufficient signal to noise ratios. Lithium doping was obtained by mixing two microliter sample with 0.5 microliter 100 mM LiCl 100 mM LiCl, one microliter of this mixture was applied on the target plate as described above.

## Additional Information

**How to cite this article**: Westereng, B. *et al.* Enzymatic cellulose oxidation is linked to lignin by long-range electron transfer. *Sci. Rep.* **5**, 18561; doi: 10.1038/srep18561 (2015).

## Supplementary Material

Supplementary Information
