# Supplementary material for: Enzymatic cellulose oxidation is linked to lignin by long-range electron transfer
Source: Sci Rep. 2015 Dec 21;5:18561. doi: 10.1038/srep18561 (PMC4685257; doi:10.1038/srep18561)
Supplement: Supplementary Information [file srep18561-s1.pdf]

Supplementary figures

Enzymatic cellulose oxidation is linked to lignin by long-range electron transfer

Bjørge Westereng[1,*,a,b], David Cannella[1,b], Jane Agger[a], Henning Jørgensen[b,d], Mogens Larsen Andersen[c], Vincent G.H. Eijsink[a], Claus Felby[b].

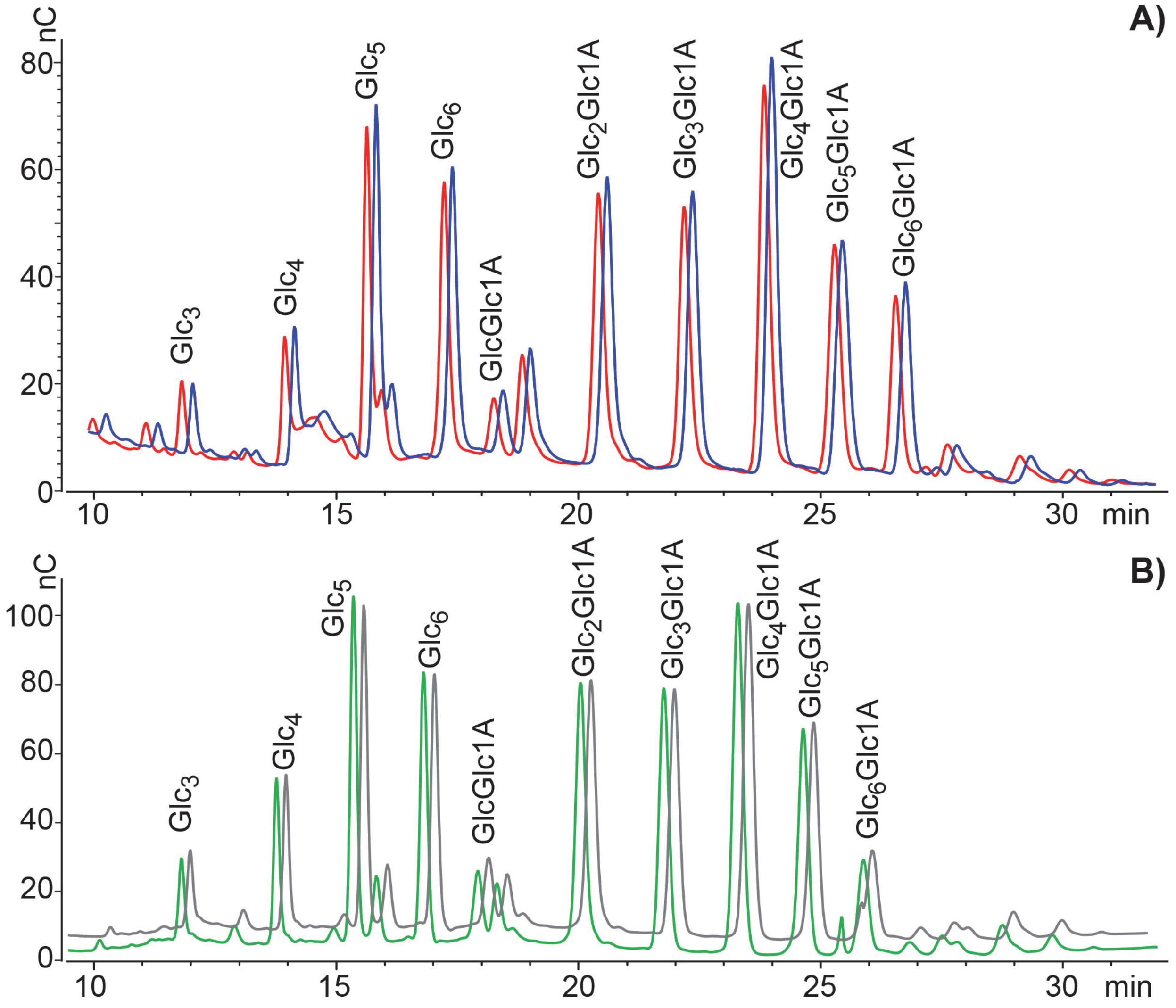

Figure S1. Synergy between high molecular weight lignin (HMWL) from pretreated wheat straw (PWS) and non-lignin compounds. A) *Tt*LPMO9E incubated with PASC and either hydroquinone (HQ, red chromatogram) or HQ+organosolv HMWL from PWS (blue

chromatogram). Samples were run in triplicates but only two chromatograms are shown for simplicity (with small time lapse). (B) *Tt*LPMO9E incubated with PASC and either 3HAA (gray chromatogram) or 3HAA and organosolv HMWL (green chromatogram). Conditions in panel A and B, (200µL total reaction volume): 1µM *Tt*LPMO9E, 50°C, 1000rpm, 0.75% PASC, 2mM reductant, and, in some reactions 1 mg HMWL; 12 hours reaction time. The highest product levels shown in this figure correspond to roughly 35 % of the maximal levels obtained after running reactions to completion.

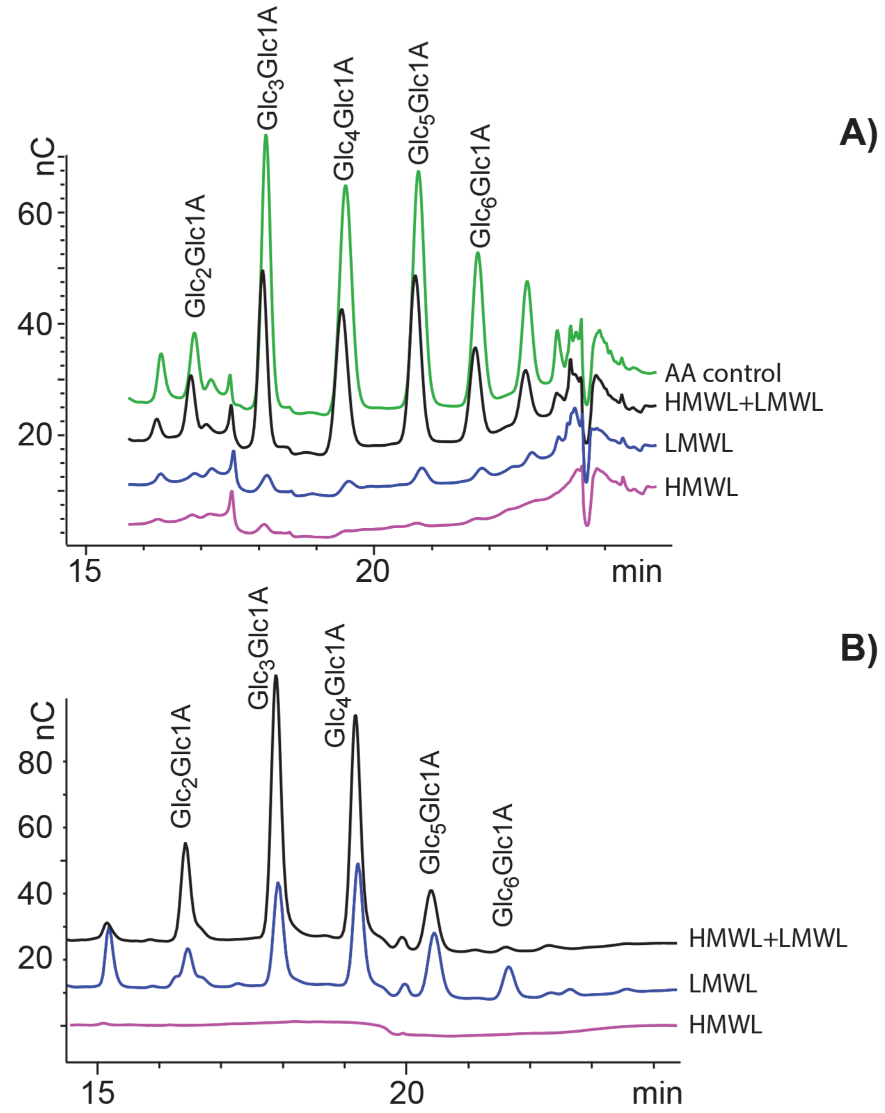

Figure S2. (A) chromatograms show C1-oxidized oligosaccharides released from PASC upon treatment with *Pc*LPMO9D in the absence of an added electron donor, but in the presence of various lignin fractions: magenta, organosolv HMWL from PWS; blue, LMWL extracted from PWS; black, mixture of LMWL and HMWL from PWS prepared by organosolv; green, control experiment with ascorbic acid (2mM), without lignin (AA). Conditions (200µL total reaction volume): 1µM *Pc*LPMO9D, 50°C, 1000rpm, 0.75% PASC, 1mg HMWL, 20µL LMWL, 12 hours reaction time. (B) Oxidized oligosaccharides released from PASC when incubated with *Pc*LPMO9D with the same conditions as in (A) but using differently prepared lignin (Klason) from PWS, showing a similar synergistic effect as in (A) when HMWL and LMWL are combined. The highest product levels shown in this figure correspond to roughly 25 % of the maximal levels obtained after running reactions to completion.
